# Amalgamation at Weymouth

**Published:** 1920-10-09

**Authors:** 


### Amalgamation at Weymouth.

An important decision has been made by the sub-committee of the Princess Christian and Royal Hospitals at Weymouth elected to consider the amalgamation proposals recently put forward. The medical men were represented by Dr. J. Macpherson Lawrie and Dr. T. H. Sanderson Wells, for the Princess Christian Hospital, and Mr. H. H. Du Boulay, for the Royal Hospital. The outcome of the joint deliberations was a unanimous decision approving of amalgamation in principle, medical and finance committees being appointed to work out the details of the scheme for subsequent adoption. The Weymouth Eye Infirmary is apparently standing out of the scheme for the present.

